# Analysis of Components and Properties of Extractives from *Alnus cremastogyne* Pods from Different Provenances

**DOI:** 10.3390/molecules27227802

**Published:** 2022-11-12

**Authors:** Guoxi Chen, Fangya Pan, Yemei Gao, Hao Li, Xiaqing Qin, Yongze Jiang, Jinqiu Qi, Jiulong Xie, Shanshan Jia

**Affiliations:** 1 College of Forestry, Sichuan Agricultural University, Chengdu 611130, China; 2 Wood Industry and Furniture Engineering Key Laboratory of Sichuan Provincial Department of Education, Chengdu 611130, China

**Keywords:** *Alnus cremastogyne* pods, extractives, UV absorbability, anti-oxidant activity

## Abstract

Chemical components with anti-oxidant, anti-inflammatory, and anti-cancer properties extracted from *Alnus* bark and leaves have been extensively studied. However, less attention has been paid to extractives from *Alnus* pods, which are mostly treated as waste. Here, extractives of *Alnus cremastogyne* pods from 12 provenances in Sichuan Province were studied for high value-added utilization of *Alnus* waste. The extractives were analyzed by Gas Chromatography-Mass Spectrometer (GC-MS), Ultraviolet-visible spectroscopy (UV-Vis spectra), and 1,1-diphenyl-2-picrylhydrazyl (DPPH) scavenging activity. A total of 58, 49, and 51 chemical components were found when the organic solvents of ethanol, petroleum ether, and ethyl acetate were used to collect extractives, respectively. These chemical components including Phytol, CIS-5,8,11,14,17-eicosapentaenoic acid, Germacrene D, Lupeol, and β-sitosterol, etc., have wide applications in the fields of pharmacy and cosmetics. Moreover, it was also found that extractives in ethanol and ethyl acetate had impressive UV resistance, especially for UV-C and UV-B blocking. The results showed that the maximum block ratio towards UV-C and UV-B could reach 99%. In addition, the ethanol extract showed good anti-oxidant activity with a maximum free radical scavenging rate of 96.19%. This comprehensive and systematic study on extractives from *Alnus cremastogyne* pods promotes the development of high-value utilization of *Alnus* components.

## 1. Introduction

As a biomass material, wood plays a very important role in human life and production. Its extractives, mainly including aliphatic compounds, terpenoid, sterpenoids, and phenolic compounds, have a strong impact on the color, durability, processing, and utilization of wood [1]. This is because wood extractives can not only provide guidance on wood classification and identification but also have a wide range of applications in the fields of medicine, food, and cosmetics, etc. [2]. *Alnus* genus belongs to *Betulaceae* Gray and widely grows in Asia, Africa, Europe, and North America, with more than 40 species. *Alnus cremastogyne* is an endemic species to China, grown in most areas of China such as Northeast, North, East, South, Central, and Southwest China [3]. Because *Alnus cremastogyne* has excellent adaptability, strong wind resistance, and smoke resistance, it is an important tree species for afforestation in many places and has high ecological value. At the same time, because of its good texture and water resistance, it can be processed as plywood, paper, musical instruments, furniture, and other materials [4,5], and has a high economic value. The bark and fruit are rich in tannins and can be used as dyes and pharmaceuticals. The leaf can be used as green fodder.

Moreover, the bark, branches, and leaves of *Alnus* can be used as a traditional medicine to treat fever, bleeding, burns, diarrhea, and alcoholism [6,7]. Chemical components of *Alnus* bark, branches, and leaves have been analyzed through extraction technology, and Diarylheptanoids, polyphenols, terpenoids, and steroids have been found [8,9,10,11]. Diarylheptanoids, as the main components of *Alnus hirsuta*, possess excellent anti-cancer and anti-oxidant properties [12]. Diarylheptanoids extracted from the bark of *Alnus glutinosa* can protect healthy cells from doxorubicin injury, and thus can be used as a protective agent for non-cancerous dividing cells during chemotherapy [13]. D. Kremer studied the reducing activity, DPPH radical scavenging activity, and B-carotene-linoleic acid anti-oxidant activity of *F. Rupestris* bark and *F. Alnus* bark. The results showed that they had good anti-oxidant and antimicrobial activities, and could be used as natural anti-oxidants, antibacterial agents, functional foods, and medicines [14]. Haoxin Li and his group found that betulin and betulinic acid, which are found in *Alnus Incana,* have good antimycobacterial activity [15]. Sun Eun Choi believed that the leaf and bark extractives of *Alnus japonica* may have some efficacy in the treatment of allergic skin diseases such as atopic dermatitis [16]. This work made significant developments in the understanding and utilization of chemical components of *Alnus*. *Alnus* is widely grown in China with high seed and pod yields, and it also has a high extractives content. However, the present research mainly focuses on *Alnus* bark, branches, and leaves. *Alnus* pods with abundant resources are usually treated as waste and the study and utilization of their extractives is paid less attention. A large number of *Alnus* pods are post-treated by landfill, incineration, or directly discarded, resulting in a great waste of resources and environmental pollution.

Here, this work thoroughly studied the extractives of *Alnus cremastogyne* pods from 12 different provenances using ethanol, petroleum ether, and ethyl acetate organic solvents. Their chemical components, anti-UV properties, and anti-oxidant properties were analyzed by Gas Chromatography-Mass Spectrometer (GC-MS), Ultraviolet–visible spectroscopy (UV-Vis spectra), and 1,1-diphenyl-2-picrylhydrazyl (DPPH) scavenging activity. We hoped to be able to analyze the chemical components of *Alnus cremastogyne* pods extractives as comprehensively as possible, and find the parts that could be used in production and utilization, so that *Alnus cremastogyne* could be fully applied to reduce waste. In addition, through the study of *Alnus cremastogyne* pod extractives in different provenances, the *Alnus cremastogyne* pod extractives and the provenances’ climate, soil, and other site conditions were linked to provide a theoretical basis for the directional cultivation of *Alnus cremastogyne*. This study provides a comprehensive and systematic understanding of the chemical components and utilization of *Alnus cremastogyne* pods, and is believed to promote the development of high-value and all-components utilization of *Alnus*.

**Figure 1:**
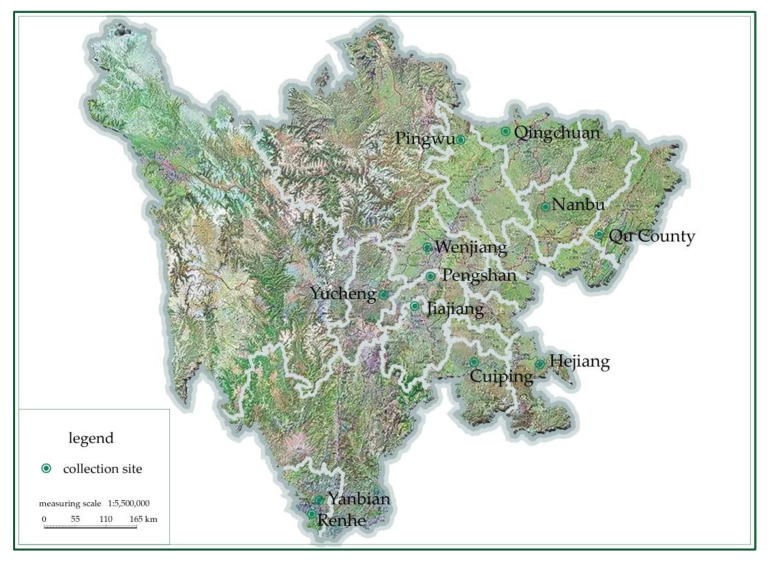
The map of different *Alnus cremastogyne* collection sites.

The pods of *Alnus cremastogyne* in this study were collected from 12 districts and counties in Sichuan Province, as shown in Table 1 and Figure 1.

## 2. Materials and Methods

### 2.1. Materials

In this study, *Alnus cremastogyne* pods from 12 different provenances were washed with deionized water and air-dried. The dried *Alnus cremastogyne* pods were broken into powder for extraction. Ethanol (≥99.9%), petroleum ether (60–90 °C), and ethyl acetate (≥99.8%) were all chromatographically pure and purchased from Knowles Company. 1,1-Diphenyl-2-picrylhydrazyl (DPPH) was purchased from Duly Biotechnology Company. Vitamin C was purchased from Sinopharm Chemical Reagent Company.

### 2.2. Organic Solvent Extraction

Five grams (accurate to 0.001 g) of completely dried *Alnus cremastogyne* pod samples were accurately weighed, wrapped in filter paper, and placed in a 250 mL Soxhlet extractor. Ethanol/petroleum ether/ethyl acetate extraction solvent was added into a 250 mL round-bottomed flask for 4 h. After cooling to room temperature, the extractives were concentrated with a rotary evaporator for use.

### 2.3. Gas Chromatograph Mass Spectrometer (GC-MS)

GC-MS analysis was performed on an 8890A gas chromatograph (Agilent, Santa Clara, CA, USA) equipped with a 5977C and mass spectrometer (Agilent, Santa Clara, CA, USA). A fused silica capillary Agilent Technology HP-5ms (5% phenyl methyl siloxane) column (30 m × 0.25 mm i.d., film thickness 0.1 μm) was used for the separation.

Analysis conditions of ethanol and ethyl acetate extractives volatile components: the injector temperature was set at 230 °C, and the detector temperature was set at 280 °C. In the first phase, the initial temperature was kept at 50 °C for 5 min, and the temperature was gradually increased to 125 °C at a rate of 3 °C/min and was then held for 3 min at 125 °C. In the second phase, the temperature was gradually increased to 180 °C at a rate of 2 °C/min and was then held for 3 min at 180 °C. In the third phase, the temperature was gradually increased to 250 °C at a rate of 15 °C/min and was then held for 8 min at 250 °C. The linear velocity of the helium carrier gas was 1 mL/min at a split ratio of 10:1. EI was used as the ion source, which was set at 200 °C. The sector mass analyzer was set to scan from 35 to 450 amu, and the scan time was set at 1 s. Extracting solution with a volume of 1.0 μL was injected for analysis.

Analysis conditions of petroleum ether extractives volatile components: the injector temperature was set at 250 °C, and the detector temperature was set at 280 °C. The initial temperature was kept at 50 °C for 4 min, and the temperature was gradually increased to 290 °C at a rate of 4 °C/min and was then held for 20 min at 290 °C. The linear velocity of the helium carrier gas was 1 mL/min at a split ratio of 10:1. EI was used as the ion source, which was set at 200 °C. The sector mass analyzer was set to scan from 10 to 600 amu, and the scan time was set at 1 s. Extracting solution with a volume of 1.0 μL was injected for analysis.

The components were identified by matching their recorded mass spectra with the standard mass spectra from the National Institute of Standards and Technology (NIST05.LIB) libraries data provided by the software of the GC-MS system, literature data, and standards of the main components.

### 2.4. Ultraviolet–Visible Spectroscopy (UV-Vis Spectra)

The optical properties of extracting solutions of ethanol, petroleum ether, and ethyl acetate of *Alnus cremastogyne* pods were measured using a UV-Vis spectrophotometer (UNICO UV-4802H) in the wavelength range 190–800 nm.

### 2.5. Radical DPPH Scavenging Activity

In this study, DPPH free radical scavenging ability was used to determine anti-oxidant capacity according to the method of Brand-Williams [17] and Sultana [18], with slight modification. The extractives were dissolved in methanol, and the sample concentration was 0.005–8 mg/mL, and the DPPH concentration was 0.04 mg/mL. Methanol was used as a blank control. The following prepared solution was reacted in the shade of room temperature for 30 min, and the absorbance was measured at 517 nm. A total of 2 mL of different concentrations of solution was added to 2 mL of DPPH solution, and the absorbance was determined as A_i_. A total of 2 mL of different concentrations of solution was added to 2 mL of methanol solution, and the absorbance was determined as A_j_. A total of 2 mL of methanol solution was added to 2 mL of DPPH solution, and the absorbance was determined as A_c_. The DPPH free radical scavenging ability SA of the sample was calculated according to the following equation:

SA(%) = [1 − (A_i_ − A_j_)/A_c_] × 100% (1)

The IC_50_ value is the effective concentration that 50% of the DPPH radical scavenged by the sample. For the measurement of the IC_50_, analysis of regression was performed to obtain the relationship between the concentration and the DPPH scavenging rate. The regression equation was calculated and the IC_50_ value obtained.

## 3. Results and Discussion

### 3.1. 100-Grain Weight and Extractives Yield

Table 2 shows the 100-grain weight of *Alnus cremastogyne* pods from 12 different provenances. It can be found that the 100-grain weight ranged from 14.15 to 49.92 g. The sample A and sample D pods were heavy with the 100-grain weight of 49.92 g and 39.26 g, while the sample L and sample E pods were light with 100-grain weights of 14.15 g and 14.45 g. According to Table 2 combined with Figure 1, there were significant differences in the 100-grain weight of pods under different site conditions. This is because sample A and sample D pods were from Ya’an and Chengdu Plain with sufficient rainfall and good soil and climatic conditions. Sample L and sample E pods were from western and southeastern Sichuan with poor soil and water conditions. The 100-grain weight of *Alnus cremastogyne* pods was affected by the soil and climate conditions. The pods in Chengdu Plain and the low hilly areas nearby were larger and heavier, while the pods in the high-mountain and hilly areas in the southeast and west of Sichuan near Yunnan-Guizhou Plateau were relatively small due to the poor soil moisture and fertilizer conditions.

The extractives yield of different samples extracted from ethanol, petroleum ether, and ethyl acetate are also presented in Table 2. The order of yield of different solvent extractives was that the yield of ethanol extractives was higher than that of ethyl acetate extractives and petroleum ether extractives, which was consistent with the order of polarity of the three solvents. The higher the polarity, the better the extraction effect. The extractives yield of ethyl acetate was from 5.20% to 17.4%, which was the highest among these three organic solvents. The extractives yield of petroleum ether and ethanol was 3.20% to 10.4%, and 5.20% to 16.8%, respectively. As for different provenances, the sample K pods had the highest extractives yield of 16.80% and 17.40% in ethanol and ethyl acetate, while the sample I pods had the highest extractives yield of 10.4% in petroleum ether. As shown in Table 2, there were also significant differences in extract yield under different site conditions. Contrary to the 100-grain weight, the extractives yield was relatively low in the regions with superior water and fertilizer conditions, because the *Alnus cremastogyne* pods grew faster and the chemical components accumulated less.

### 3.2. GC-MS Analysis

**Figure 2:**
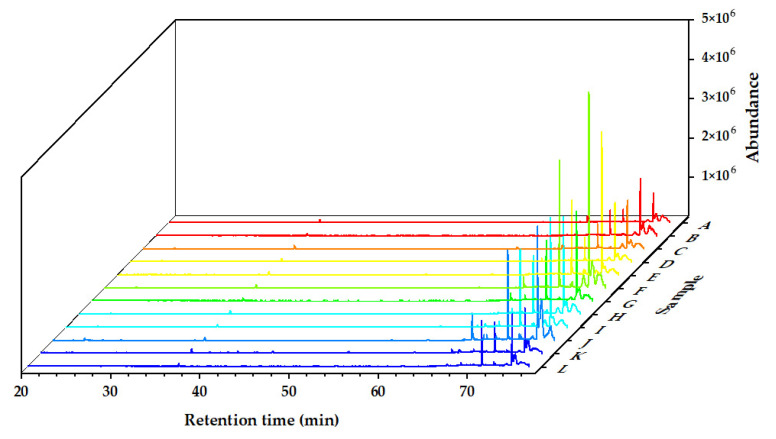
GC-MS chromatograms of the volatile components of the ethanol extractives of *Alnus cremastogyne* pods.

**Figure 3:**
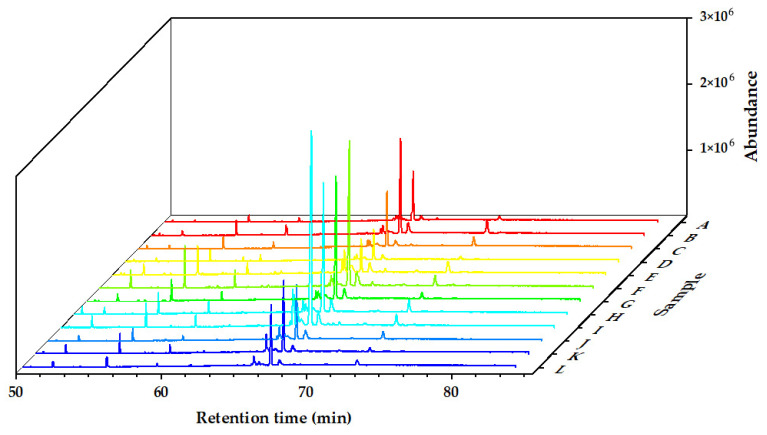
GC-MS chromatograms of the volatile components of the petroleum ether extractives of *Alnus cremastogyne* pods.

**Figure 4:**
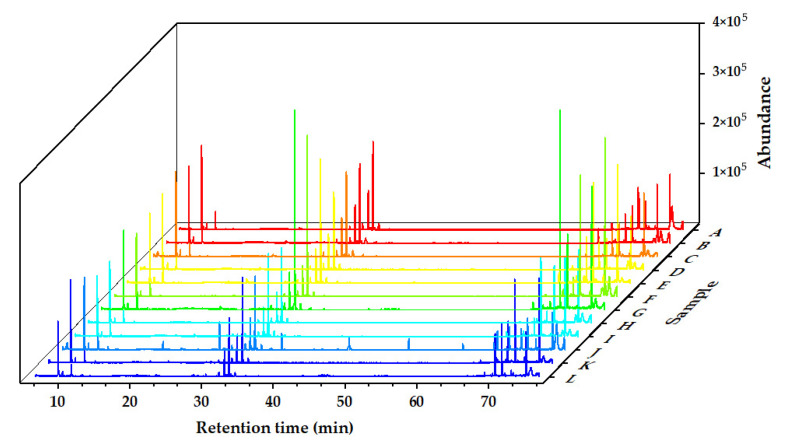
GC-MS chromatograms of the volatile components of the ethyl acetate extractives of *Alnus cremastogyne* pods.

The chemical components of the *Alnus cremastogyne* pod extractives’ volatile components were identified by GC-MS. Figure 2, Figure 3 and Figure 4 show the total ion chromatograms of the volatile components of different extractives. In the chromatogram of ethanol extractives, an obvious Eicosane characteristic peak appeared at the retention time of 74.3 min (Figure 2), and in the chromatogram of petroleum ether extractives, the characteristic peak of Lup-20(29)-en-3-one appeared at the retention time of 67.2 min (Figure 3). Around 29.9 min, the characteristic peak of Isoledene appeared in the ethyl acetate extractives (Figure 4). The above characteristic peaks can be used as a reference for *Alnus cremastogyne* identification. 

The identified chemical components are shown in Table 3, Table 4 and Table 5. A total of 59, 49, and 51 chemical components were identified around when ethanol, petroleum ether, and ethyl acetate were used as the organic solvent, respectively. Table 3, Table 4 and Table 5 show that the main chemical components of the three solvent extractives of *Alnus cremastogyne* pods were alkenes, ketones, carboxylates, ethers, benzenes, esters, and a small number of halides and complex compounds. Table 3 shows that the ethanol extractives were mainly chemical components of Phenol, 3,5-bis(1,1-dimethylethyl)-, 1*R*,4*S*,7*S*,11*R*-2,2,4,8-Tetramethyltricyclo[5.3.1.0(4,11)] undec-8-ene, Nonadecane, 6*β*-Bicyclo[4.3.0] nonane, 5β-iodomethyl-1β-isopropenyl-4α.,5α.-dimethyl-, Octadecane, and Eicosane. As shown in Table 4, for petroleum ether extractives, the main chemical components were Phenol, 2,2’-methylenebis[6-(1,1-dimethylethyl)-4-methyl-, Undecane, 3,8-dimethyl-, D-Friedoolean-14-en-3-one, A’-Neogammacer-22(29)-en-3-one, 13,27-Cycloursan-3-one, γ-Sitosterol, Lup-20(29)-en-3-one, Lupeol, and Eicosane, 1-iodo-. As for ethyl acetate extractives (Table 5), the main constituents were Bicyclo[3.1.0]hex-2-ene, 4-methyl-1-(1-methylethyl)-, Camphene, Ethanol, 1,1’-oxybis-, diacetate, α-Cubebene, 1,3-Cyclohexadiene, 5-(1,5-dimethyl-4-hexenyl)-2-methyl-, [S-(R*,S*)]-, (1*R*,5*R*)-2-Methyl-5-((*R*)-6-methylhept-5-en-2-yl)bicyclo[3.1.0]hex-2-ene, β-Gurjunene, isoledene, (*E*)-β-Famesene, Phytol, Eicosane, 1-iodo-, Phenol, 2,2’-methylenebis[6-(1,1-dimethylethyl)-4-methyl-, Bicyclo[3.1.1]heptane, 6,6-dimethyl-2-methylene-, (1*S*)-, cis-Muurola-4(15),5-diene, cis-muurola-3,5-diene, Di-n-decylsulfone, Eicosane, 7-hexyl-, Undecane, 3,8-dimethyl-, and A’-Neogammacer-22(29)-en-3-one. Many of these chemical components have been studied and utilized in the pharmaceutical, cosmetic, and food industries.

In the ethanol extractives, Phytol has anti-injury, anti-oxidant, anti-inflammatory and anti-allergic effects. It also has antibacterial activity against Mycobacterium tuberculosis and Staphylococcus aureus [19,20]. Cis-5,8,11,14,17-eicosapentaenoic acid can reduce blood viscosity, reduce thrombosis, improve serum, and reduce blood sugar, and also has cerebrovascular protection, anti-allergy, and anti-cancer effects [21]. Picrotoxinin is a potent convulsant, and a noncompetitive GABAA receptor antagonist that negatively modulates the effects of GABA on GABAA receptors [22]. Germacrene D as a precursor plays an important role in sesquiterpene biosynthesis [23]. Caryophyllene oxide in clinical trials shows good central and peripheral analgesic and anti-inflammatory activity, and it can be used for the treatment of onychomycosis. It also has antifungal properties and is widely used as a preservative in cosmetics and daily necessities [24,25]. Other chemical components such as Butanoic acid, 2-methyl-, Decanoic acid, ethyl ester, Citronellyl isobutyrate, Nonanoic acid, 5-methyl-, ethyl ester, 2H-Pyran-2-One, Tetrahydro-6-nonyl-, and so on, can be used as raw materials as a configuration of a variety of fruit-based flavors, added to a variety of common food and drinks.

In petroleum ether extractives, existing studies have found that Squalene is effective on hypoxia resistance and thus can be widely used in the domestic market. Because Squalene possesses a stronger anti-oxidant capacity compared with other lipid molecules in the skin, it can help the skin resist damage caused by UV irradiation and other oxidation reactions. It has also been reported that Squalene has moisturizing properties, and it is widely used in creams, lotions, hair creams, lipsticks, and other cosmetics [26,27,28]. Lupeol has anti-oxidant, anti-inflammatory, and skin-healing properties. It also has an inhibitory effect on breast cancer, prostate cancer, and melanoma [29,30]. β-sitosterol can reduce cholesterol, act as an anti-diabetic cough expectorant, inhibit tumors, and repair tissue [31,32]. Octacosanal has obvious biological activities on both humans and animals. It has physiological functions such as fatigue, metabolism promotion, anti-atherosclerosis, and prevention and treatment of Parkinson’s disease and Alzheimer’s disease. It is widely used in medicine, food, cosmetics, feed, and other fields [33,34,35]. Betulinaldehyde and Betulin also have anti-tumor, anti-inflammatory, and anti-allergic effects, which can be used in content determination, identification, and pharmacological experiments [36,37]. In addition to the pharmaceutical effects of the above-mentioned ingredients, 1H-tetrazol-5-amine, Nonadecane, Pentacosane, Hexadecane, and 3, 5-dimethoxycinnamic acid can be used as raw materials for chemical analysis and pharmaceutical synthesis.

In the ethyl acetate extractives, divided by the mentioned Germacrene D, Phytol, and other chemical components, Camphene with good anti-oxidant and cholesterol-reducing properties, can also be used as a raw material for the synthesis of camphor, and spices [38,39]. 1-Adamantanemethylamine, α-methyl- has a good therapeutic and inhibitory effect on influenza A, and can be used in the manufacture of various kinds of levofloxacin capsules, tablets, and other anti-microbial preparations [40,41,42]. γ-sitosterol has anti-hyperglycemia and lipid-lowering effects and can be used as an effective lipid-lowering agent for the treatment of hyperglycemia [43,44]. Cyclobarbital is a type of barbiturate derivative that has sedative and hypnotic effects and can be used as a surgical anesthetic [45].

### 3.3. UV Absorbability

**Figure 5:**
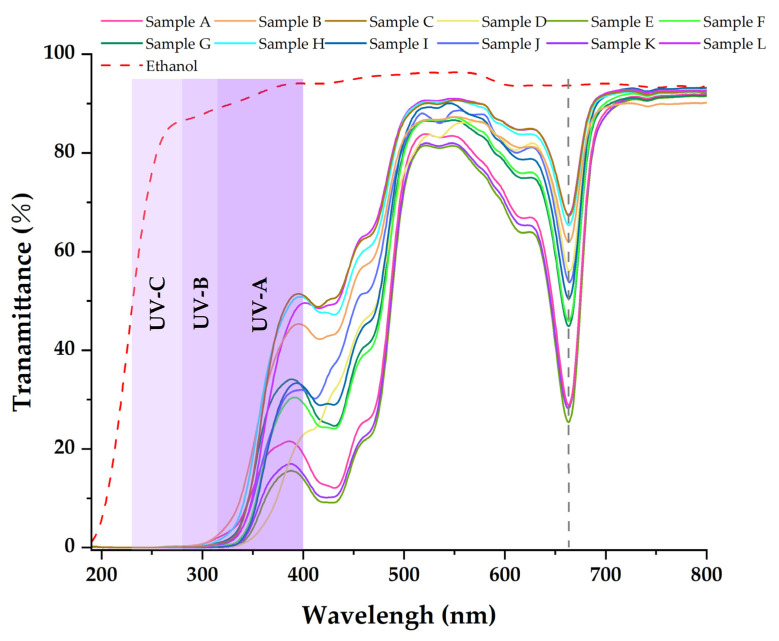
Transmittance spectra of the ethanol extractives from *Alnus cremastogyne* pods.

**Figure 6:**
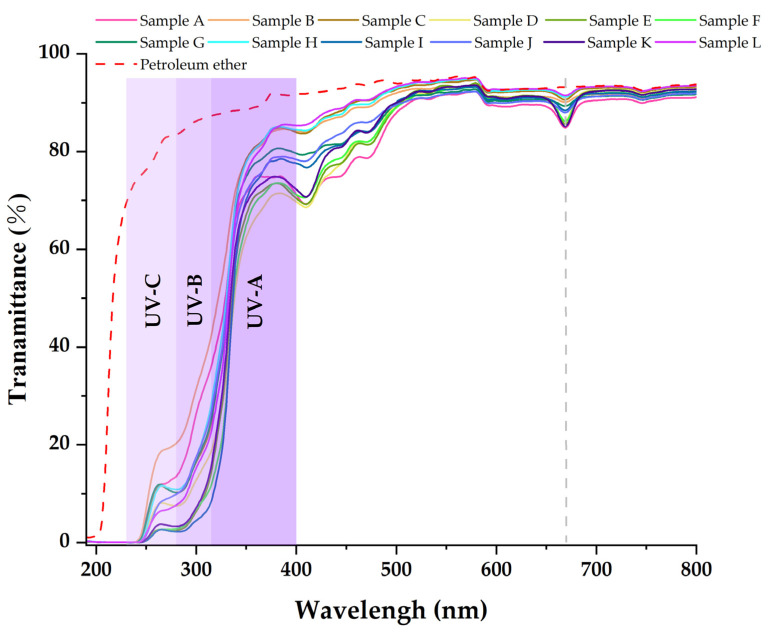
Transmittance spectra of the petroleum ether extractives from *Alnus cremastogyne* pods.

**Figure 7:**
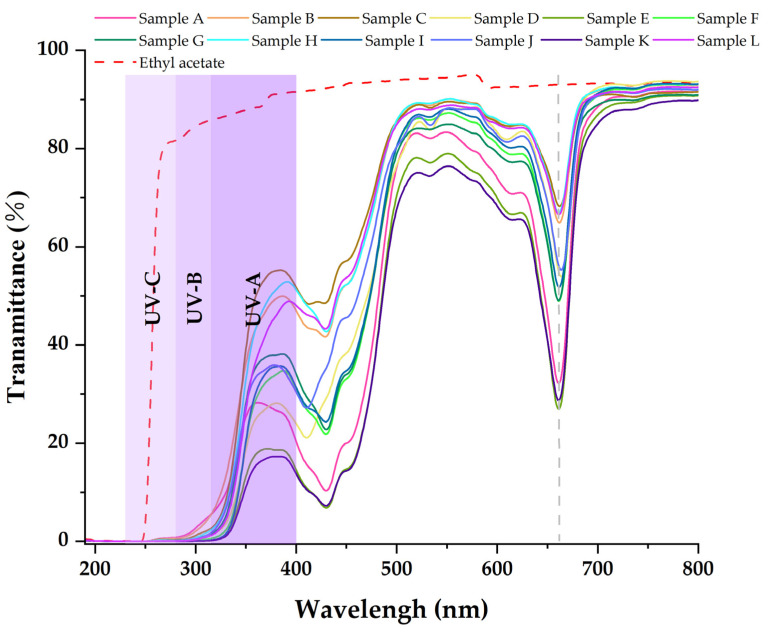
Transmittance spectra of the ethyl acetate extractives from *Alnus cremastogyne* pods.

Table 3, Table 4 and Table 5 show that the main chemical components of the three solvent extractives of *Alnus cremastogyne* pods are olefin, ketones, carboxylates, ethers, benzenes, esters, and a small number of halides and complex compounds. Most of these substances have good UV absorption capacity, especially olefin, ketones, and benzene compounds. As shown in Figure 5, Figure 6 and Figure 7, in the visible spectrum, the 12 samples in near 665 nm wavelength visible light peak, it may be because the extraction process also extracts the chlorophyll A components from the *Alnus cremastogyne* pods. The peak values of the ethyl acetate and ethanol extracting solutions were lower than those of the petroleum ether extracting solution, which may be due to their greater polarity and better extraction effect. In the UV light band, the absorbability of the three solvent control samples in the UV-A (315–400 nm) and UV-B (280–315 nm) bands was lower than 20% and increased in the UV-C (230–280 nm) and below bands. In general, the three solvents had poor UV absorbability, but three kinds of solvent extracting solution of 12 samples showed good UV absorbability, especially the UV-C (230–280 nm) and UV-B (280–315 nm) bands. Three kinds of solvent extracting solution ultraviolet transmittance were all less than 50%, the ethanol extracting solution in the UV-B (280–315 nm) band absorbability was greater than 96%, and the ethyl acetate extracting solution absorbability was more than 94%. The absorbability of ethanol and ethyl acetate was more than 99% in the UV-C (230–280 nm) band. In the UV-A (315–400 nm) band, the ethanol extracting solution has the best absorbability, which absorbed almost more than 50%–85% of the UV-A (315–400 nm) band, followed by the ethyl acetate extracting solution, which absorbed almost more than 45%–80% of the UV-A (315–400 nm) band. The petroleum ether extracting solution had poor absorbability in the UV-A (315–400 nm) band. It only absorbed 10%–30% around the wavelength of 385 nm, which may have been related to the composition and content of the extractives that absorb UV. For example, the benzene substances in the absorbability of ethanol and ethyl acetate were significantly more than those of petroleum ether, while the ethanol extractives were more than that of ethyl acetate extractives. In addition, the low UV absorbability in the UV-A (315–400 nm) band may have been caused by the presence of conjugated bonds in the chemical components, which made the UV absorption peak of -S=O, -S-O, C_6_H_6_, COO-, -O-, C=O, -Coor and other chromogenic functional groups in the compound red-shift, thus reducing the UV absorbability of the solution in this band. These studies showed that the ethanol and ethyl acetate extractives of *Alnus cremastogyne* pods had good UV absorbability, which is valuable for research, and they could be used in strong UV protection products after separation and purification.

### 3.4. Anti-Oxidant Activity

The anti-oxidant activity was evaluated by measuring the scavenging ability of the *Alnus cremastogyne* pod extractives against DPPH free radicals. As Table 6 shows, the scavenging ability of the extracting solution against DPPH radicals increased with the increase of the concentration of the extracting solution. By calculating the concentration of extracting solution when the DPPH free radical scavenging rate of extractives was 50% (IC_50_), the anti-oxidant activity of *Alnus cremastogyne* pods extractives from different provenances of three solvents was compared. As Table 6 shows, when the DPPH free radical scavenging rate of ethanol extracting was 50%, the concentration of the extracting solution was the lowest (0.076 mg/mL–1.992 mg/mL), and the anti-oxidant activity was the best. When the concentration of petroleum ether extracting solution was the highest (1.518 mg/mL–5.265 mg/mL), the anti-oxidant activity was the worst. The IC_50_ value of ethyl acetate was 0.349 mg/mL to 3.283 mg/mL. This is related to the amount of -OH ions in the compound.

In extractives of *Alnus cremastogyne* pods from 12 different provenances. The IC_50_ values of ethanol, petroleum ether, and ethyl acetate extractives of sample D and sample J were lower than those of the other 10 provenances, indicating that they had better anti-oxidant activity. The IC_50_ values of the ethanolic extractives of sample D and sample J were only 0.076 mg/mL and 0.184 mg/mL, and the DPPH free radical scavenging rates of 0.5mg/mL ethanolic extractives reached 95.79% and 90.35%; DPPH free radical scavenging rate can be up to 95.91% and 96.19%. It has a high anti-oxidant activity that is higher than tea extractives [46], Essential Oil [47], Oilseeds [48], and other plants and foods. The IC_50_ value of sample D is more than 30 times higher than that of Vitamin C, but it may have a high anti-oxidant potential for further isolation and purification in later studies. These studies indicate that the anti-oxidant activity of *Alnus cremastogyne* pod extractives is valuable to be studied and utilized. *Alnus cremastogyne* pods can also be used as a raw material, along with *Alnus cremastogyne* bark, branches, and leaves, to extract the active ingredients of anti-inflammatory and anti-cancer drugs.

## 4. Conclusions

The results show that the volume and weight of *Alnus cremastogyne* pods are closely related to the site conditions. The pods are larger and heavier in areas with superior climate and soil conditions, and smaller in areas near the plateau due to poor soil and water conditions. The same solvent extractives’ yield rules are different, which may be the pods’ growth speed and less chemical accumulation. This study on *Alnus cremastogyne* pod extractives can be used to guide the directed cultivation of *Alnus cremastogyne*.

A total of 59, 49, 51 chemical components were found when the organic solvents of ethanol, petroleum ether, and ethyl acetate were used to collect extractives. Many chemical components have anti-oxidant, anti-inflammatory, and other effects that can be used in pharmaceuticals, cosmetics, and other fields. The ethanol extractives of *Alnus cremastogyne* pods show good performance in UV absorbability and anti-oxidant activity analysis and can be further separated and purified as natural raw materials for anti-UV and anti-oxidant products. The ethanol extractives have higher relative content, the most types of chemical components, the best ability of UV absorption and anti-oxidant activity, and have the value of development and utilization. In the future, through the directional cultivation of *Alnus cremastogyne*, its pod extractives have great application potential in the fields of biomedicine, fine chemical intermediates, light industry and food, health care products, and cosmetics.

## Figures and Tables

**Table 1:** Information on different *Alnus cremastogyne* collection sites.

Sample	Age (Year)	Provenances	Latitude and Longitude
A	20	Yucheng District, Ya’an City	E 103°01′20″ N 29°58′44″
B	21	Yanbian County, Liangshan Prefecture	E 102°34′23″ N 26°37′43″
C	18	Pingwu County, Mianyang City	E 104°32′02″ N 32°24′38″
D	21	Wenjiang District, Chengdu City	E 103°47′32″ N 30°44′36″
E	23	Hejiang County, Luzhou City	E 105°49′10″ N 28°48′48″
F	21	Qingchuan County, Guangyuan City	E 105°14′38″ N 32°34′59″
G	20	Qu County, Dazhou City	E 106°57′28″ N 30°50′05″
H	20	Nanbu County, Nanchong City	E 105°48′00″ N 31°15′12″
I	19	Pengshan District, Meishan City	E 103°49′40″ N 30°17′03″
J	18	Cuiping District, Yibing City	E 104°39′46″ N 28°52′11″
K	21	Jiajiang County, Leshan City	E 103°33′44″ N 29°45′09″
L	20	Renhe District, Panzhihua City	E 101°45′10″ N 26°28′50″

**Table 2:** 100-grain weight and extractives yield of the *Alnus cremastogyne* pod extractives.

Sample	100-Grain Weight(g)	Extractive Yield		
		Ethanol	Petroleum Ether	Ethyl Acetate
A	49.92 ± 1.02 ^a^	6.40 ± 0.20% ^h^	4.20 ± 0.30% ^a^	7.60 ± 0.60% ^f^
B	17.07 ± 0.34 ^i^	7.20 ± 0.50% ^f^	4.80 ± 0.20% ^c^	6.80 ± 0.40% ^g^
C	30.05 ± 0.61 ^d^	7.80 ± 0.60% ^f^	5.20 ± 0.40% ^b c^	8.20 ± 0.50% ^f^
D	39.26 ± 0.45 ^b^	5.20 ± 0.30% ^i^	3.20 ± 0.20% ^b c^	5.20 ± 0.30% ^g^
E	14.45 ± 0.22 ^j^	11.40 ± 0.40% ^c^	7.00 ± 0.50% ^b c^	12.80 ± 0.40% ^c^
F	17.45 ± 0.30 ^i^	10.00 ± 0.80% ^d^	6.60 ± 0.40% ^c d^	12.00 ± 0.60% ^d^
G	21.94 ± 0.41 ^h^	10.40 ± 0.60% ^d^	7.00 ± 0.40% ^d e^	11.40 ± 0.70% ^d^
H	23.42 ± 0.55 ^f^	11.60 ± 0.60% ^c^	7.00 ± 0.60% ^e^	11.20 ± 0.50% ^d e^
I	34.76 ± 1.25 ^c^	12.80 ± 0.50% ^b^	10.40 ± 0.60% ^e f^	15.40 ± 0.20% ^b^
J	26.35 ± 0.47 ^e^	9.00 ± 0.20% ^e^	6.00 ± 0.30% ^f g^	8.60 ± 0.30% ^f^
K	34.00 ± 1.01 ^c^	16.80 ± 0.40% ^a^	7.60 ± 0.40% ^g^	17.40 ± 0.40% ^a^
L	14.15 ± 0.29 ^j^	9.00 ± 0.30% ^e^	5.40 ± 0.50% ^h^	10.00 ± 0.50% ^c^

Different letters following the mean values in the same column mean significant difference at 0.05 level (*p* < 0.05).

**Table 3:** Chemical compositions of the ethanol extractives of *Alnus cremastogyne* pods.

S/N	Compound	Relative Amount (%)											
		A	B	C	D	E	F	G	H	I	J	K	L
1	Phenol, 3,5-bis(1,1-dimethylethyl)-	12.02	5.25	7.01	6.60	3.20	2.18	4.28	3.88	2.90	2.59	11.41	6.75
2	Sulfurous acid, 2-ethylhexyl isohexyl ester	0.67					2.17	2.88	2.33	2.28		2.57	
3	Undecanoic acid, ethyl ester	3.86	1.39										
4	Phytol	1.62		1.08				0.75		0.84	0.68		0.72
5	1,9-Cyclohexadecadiene	13.78						4.98					
6	1-Tetradecyne	3.26					0.74						
7	1-Octadecanesulphonyl chloride	1.39	0.64	3.76		0.52							2.47
8	Phenol, 2,2’-methylenebis[6-(1,1-dimethylethyl)-4-methyl-	5.64	1.10	1.79	3.12			1.04	0.81			1.84	1.09
9	Dodecane, 2,6,11-trimethyl-	29.29											
10	1*R*,4*S*,7*S*,11*R*-2,2,4,8-Tetramethyltricyclo[5.3.1.0(4,11)]undec-8-ene	23.42	18.53	25.68		10.44		10.92				10.50	2.84
11	Nonadecane		30.08	34.58		39.60		46.66	41.68	43.81			59.27
12	6β-Bicyclo[4.3.0]nonane, 5β-iodomethyl-1β-isopropenyl-4α,5α-dimethyl-,	30.61	23.99	53.26	22.85	4.91	35.98	21.93	31.78		42.17	88.54	59.91
13	Card-20(22)-enolide, 3,5,14,19-tetrahydroxy-, (3β,5β)-	90.58											
14	Azulene, 1,2,3,5,6,7,8,8a-octahydro-1,4-dimethyl-7-(1-methylethenyl)-, [1*S*-(1α,7α,8aβ)]-	45.35				8.06							
15	9,12-Octadecadienoic acid, methyl ester, (*E*,*E*)-		1.08								12.06		0.90
16	7-Oxabicyclo[4.1.0]heptane, 1-methyl-4-(2-methyloxiranyl)-		2.89			1.25				0.65		0.85	1.59
17	3,4-Hexanedione, 2,2,5-trimethyl-		3.15		0.70								
18	Cis-5,8,11,14,17-eicosapentaenoic acid		0.57										
19	2,5,5,8a-Tetramethyl-4-methylene-6,7,8,8a-tetrahydro-4H,5H-chromen-4a-yl hydroperoxide		1.64	1.24							1.27		
20	Octacosane		3.16										
21	Octadecane										80.85		
22	trans-Z-α-Bisabolene epoxide		62.46									5.72	0.53
23	1-Cycloheptene, 1,4-dimethyl-3-(2-methyl-1-propene-1-yl)-4-vinyl-		28.60		61.73				17.54				
24	Butanoic acid, 2-methyl-			0.55	3.60							1.39	
25	Dodecanoic acid, ethyl ester			5.56						1.37		3.04	
26	9,12-Octadecadienoyl chloride, (*Z*,*Z*)-			1.72					0.53	3.11			3.21
27	β-Guaiene			51.87									
28	Retinal				3.11								
29	Decanoic acid, ethyl ester				1.52	0.55	0.52	0.89			0.96		2.00
30	Citronellyl isobutyrate				6.35								
31	Linoelaidic acid				0.81								
32	Hexane, 3,3-dimethyl-				1.65	1.64	0.60	0.73	2.86		1.88	2.30	2.64
33	Hexadecane				30.61	2.04	2.46			2.33	2.06	44.83	
34	Diepicedrene-1-oxide				1.06				1.34	1.61			4.45
35	Dodecane, 1-iodo-				4.02								
36	Thunbergol				25.40					22.23	5.15		
37	(1*R*,6*S*)-6-Hydroxy-6-methyl-4-oxocyclohex-2-en-1-yl benzoate					0.69				0.75		2.43	1.57
38	2,12-Dimethylidenecyclododecan-1-one					2.46					2.37		
39	Eicosane	100.00	100.00	100.00	100.00	100.00	100.00	100.00	100.00	100.00	100.00	100.00	100.00
40	(*Z*)6,(*Z*)9-Pentadecadien-1-ol						3.88				1.63		
41	Aromadendrene oxide-(1)						1.56	0.98				3.20	
42	Humulane-1,6-dien-3-ol						19.66						
43	Heptacosane						43.29						
44	β-Sitosterol						10.67						
45	1H-Benzocycloheptene, 2,4a,5,6,7,8,9,9a-octahydro-3,5,5-trimethyl-9-methylene-, (4a*S*-cis)-						25.79						
46	2-Isopropenyl-4a,8-dimethyl-1,2,3,4,4a,5,6,8a-octahydronaphthalene										22.27		
47	Diazoprogesterone							7.25					
48	Naphthalene, decahydro-4a-methyl-1-methylene-7-(1-methylethenyl)-, [4a*R*-(4aα,7α,8aβ)]-							5.97					
49	α-Guaiene								52.78	18.89			
50	Alloaromadendrene oxide-(1)									31.94			
51	Nonanoic acid, 5-methyl-, ethyl ester												0.50
52	1H-Cycloprop[e]azulene, 1a,2,3,5,6,7,7a,7b-octahydro-1,1,4,7-tetramethyl-, [1a*R*-(1aα,7α,7aβ,7bα)]-												10.94
53	Picrotoxinin												8.78
54	(1*R*,2*R*,4*S*,6*S*,7*S*,8*S*)-8-Isopropyl-1-methyl-3-methylenetricyclo[4.4.0.02,7]decan-4-ol											4.88	
55	(1*R*,7*S*,*E*)-7-Isopropyl-4,10-dimethylenecyclodec-5-enol											6.54	
56	1,14-Tetradecanediol											7.28	
57	2H-Pyran-2-one, tetrahydro-6-nonyl-											0.64	
58	Caryophyllene oxide											0.93	
59	Aromadendrene oxide-(2)											43.4	

**Table 4:** Chemical compositions of the petroleum ether extractives of *Alnus cremastogyne* pods.

S/N	Compound	Relative Amount (%)											
		A	B	C	D	E	F	G	H	I	J	K	L
1	Phenol, 2,2’-methylenebis[6-(1,1-dimethylethyl)-4-methyl-	1.85	1.09	2.71	3.82	0.50			3.38		0.82	1.48	0.53
2	1H-Tetrazol-5-amine	2.93											
3	Undecane, 3,8-dimethyl-	11.68	6.78	7.31	13.90	25.16		4.61				7.91	
4	Squalene	1.22					0.90		0.70		1.34		
5	Hexane, 3,3-dimethyl-	5.81		3.54									
6	D-Friedoolean-14-en-3-one	2.98	4.33	9.14	7.64	35.29	1.11	4.91	7.07	1.37	5.38	1.80	2.03
7	A’-Neogammacer-22(29)-en-3-one	6.22	22.18	11.58	17.67	68.56	8.96	6.74	13.13	11.95	18.66	24.46	12.55
8	13,27-Cycloursan-3-one	7.61	8.45	27.29	19.58	50.25	2.07	3.12	2.71	8.01	22.81	7.34	4.07
9	γ-Sitosterol	4.19	0.67	4.06	13.72	20.60	3.72	6.35	5.48		16.22	9.03	
10	Olean-18-ene	7.00											
11	Lup-20(29)-en-3-one	100.00	100.00	100.00	100.00	100.00	100.00	100.00	100.00	100.00	100.00	100.00	100.00
12	Lupeol	16.74	19.86	19.22	26.80	54.82	16.91	15.76	16.85	9.58	30.74	17.63	17.39
13	γ-Sitostenone	3.60		3.95									
14	β-Amyrone	11.97								5.91			13.02
15	Octane, 2,7-dimethyl-		3.01						5.97	2.67			11.01
16	Nonadecane		10.57										
17	β-Sitosterol		2.09			13.28							
18	6a,14a-Methanopicene, perhydro-1,2,4a,6b,9,9,12a-heptamethyl-10-hydroxy-		3.39		15.83			6.28	6.70	4.57			
19	17α,21β-28,30-Bisnorhopane		2.80			3.94	0.54		0.50	0.65			
20	Dodecane, 2,6,11-trimethyl-		13.73										
21	Eicosane, 1-iodo-			0.76	29.55	2.36	6.57			2.92	14.38		
22	Germanicol			14.32		40.70					9.02	9.10	2.99
23	Decane, 2,4-dimethyl-				7.61	2.35							
24	Di-n-decylsulfone				0.86	3.62						0.72	
25	Heptacosane				1.86		0.64					18.57	
26	3,7-Dimethyl-1-phenylsulfonyl-2,6-octadiene				9.58								
27	Pentacosane						6.59	10.30	9.70	5.83			
28	Hexacosane						15.53						
29	α-Amyrone					1.12	3.79			0.58			
30	9,19-Cyclolanost-23-ene-3,25-diol, (3β,23*E*)-							4.67	4.42	1.13			
31	Undecane, 4,8-dimethyl-												6.41
32	Sulfurous acid, 2-ethylhexyl isohexyl ester												3.94
33	Octacosanal					6.26	1.06		0.74			1.92	1.55
34	9,19-Cyclo-27-norlanostan-25-one, 3-(acetyloxy)-24-methyl-, (3β,24*R*)-									6.35			4.25
35	Undecane, 3,7-dimethyl-											8.63	
36	Stigmast-4-en-3-one					11.69	2.06	0.86				2.22	
37	7a-Isopropenyl-4,5-dimethyloctahydroindene-4-carboxylic acid											3.77	
38	Decane, 2,3,5-trimethyl-								3.52				
39	Hexadecane							3.49					
41	Decane, 2,9-dimethyl-										5.84		
42	Nonane, 3,7-dimethyl-										5.66		
43	3,5-Dimethoxycinnamic acid										1.45		
44	Spirost-8-en-11-one, 3-hydroxy-, (3β,5α,14β,20β,22β,25*R*)-										2.56		
45	3,4-Hexanedione, 2,2,5-trimethyl-					3.48							
46	Octatriacontyl pentafluoropropionate					4.56							
47	3,6,9,12-Tetraoxatetradecan-1-ol, 14-[4-(1,1,3,3-tetramethylbutyl)phenoxy]-					2.34							
48	Betulinaldehyde					4.10							
49	Betulin					9.75							

**Table 5:** Chemical compositions of the petroleum ether extractives of *Alnus cremastogyne* pods.

S/N	Compound	Relative Amount (%)											
		A	B	C	D	E	F	G	H	I	J	K	L
1	Bicyclo[3.1.0]hex-2-ene, 4-methyl-1-(1-methylethyl)-	71.19	69.59	67.17	68.05	44.44	31.88	50.81	68.78		58.44	56.97	73.08
2	Camphene	5.84	5.25	5.66	5.56	3.56	2.39	4.12	5.12	4.58	4.56	4.26	5.28
3	Bicyclo[3.1.0]hexane, 4-methylene-1-(1-methylethyl)-	15.40		14.38			6.11			12.60			
4	Ethanol, 1,1’-oxybis-, diacetate	4.40	4.00	5.27	3.29	6.08	2.32	4.20				3.62	
5	Germacrene D	1.07		1.68					1.40	1.17		6.60	
6	α-Cubebene	3.22		3.05		2.01	1.19		2.72		2.57		
7	(1*R*,5*R*)-2-Methyl-5-((*R*)-6-methylhept-5-en-2-yl)bicyclo[3.1.0]hex-2-ene	7.72	7.44	7.15	6.91	4.71	3.07		6.83	5.40	0.68	4.95	5.96
8	β-Gurjunene	44.96	44.33	42.7	43.34	28.67	19.94	31.81	43.11	37.92	35.4	33.57	42.00
9	isoledene	100.00	100.00	100.00	100.00	100.00	100.00	100.00	100.00	100.00	100.00	100.00	100.00
10	cis-β-Farnesene	10.03	10.43		1.55	0.83	4.28	8.93		9.96	8.34		8.94
11	(*E*)-β-Famesene	1.26	1.62	1.46	10.16		0.51	1.05	1.06	1.17	1.19		1.08
12	Phytol	8.39	21.47	1.72	4.36		1.02	6.44	1.41	5.36	7.30	2.66	
13	Eicosane, 1-iodo-	0.86	2.23				2.34	0.77	2.53	3.38	0.90	1.87	2.36
14	Phenol, 2,2’-methylenebis[6-(1,1-dimethylethyl)-4-methyl-	24.78	17.93	16.31	21.06	19.05	6.00	87.51	8.89	12.14	10.41	26.11	35.49
15	Hexane, 3,3-dimethyl-	21.25									22.21		
16	Nonane, 3,7-dimethyl-	59.89											68.42
17	16-Hentriacontanone	89.14											
18	γ-Sitosterol	2.27					16.35						
19	Bicyclo[3.1.1]heptane, 6,6-dimethyl-2-methylene-, (1*S*)-		15.19		14.29	9.10		10.64			13.40	11.71	
20	cis-muurola-4(15),5-diene		1.62		1.83		0.52		0.80	2.30	0.95	2.07	3.11
21	cis-muurola-3,5-diene		3.05		2.87	0.58				0.87		0.63	2.85
22	Bicyclo[3.1.1]heptane, 6-methyl-2-methylene-6-(4-methyl-3-pentenyl)-, [1R-(1α,5α,6β)]-		2.08	2.01	2.17				1.85	1.62			
23	Cubenene		1.72					2.35					
24	Di-n-decylsulfone		1.48	1.78	1.23	2.79	0.67			1.78	0.86	3.15	3.41
25	Sulfurous acid, 2-ethylhexyl isohexyl ester		30.28										
26	Eicosane, 7-hexyl-		3.51			6.13	2.07			2.82			1.52
27	Ginsenol		11.70	7.14		2.34						3.04	
28	Undecane, 3,8-dimethyl-		71.11	72.59	65.88			41.76	52.02	85.15		73.22	
29	A’-Neogammacer-22(29)-en-3-one		44.50	18.37	25.20	11.65	41.88	24.64	39.50	23.36	76.29	25.47	44.12
30	Germanicol		8.83								22.69		
31	Butanoic acid, 2-methyl-			6.18							13.89		
32	Cyclohexene, 3-(1,5-dimethyl-4-hexenyl)-6-methylene-, [S-(R*,S*)]-			9.84		6.43		7.48				0.69	
33	Sulfurous acid, 2-ethylhexyl hexyl ester			25.62	26.61								
34	D-Homopregn-17a(20)-ene, (5α,17a*E*)-				28.44								
35	Nonadecane					64.86	50.27						
36	6βBicyclo[4.3.0]nonane, 5β-iodomethyl-1β-isopropenyl-4α,5α-dimethyl-,					4.22	2.01					4.42	5.60
37	5α-Pregn-16-en-20-one						13.28						
38	Pentacosane						44.58						
39	Tetradecane, 1-iodo-					55.35							
40	Hexadecane								97.11	66.09	39.65	60.54	54.48
41	Methyl 3-bromo-1-adamantaneacetate											7.16	
42	Cyclohexene, 4-methylene-1-(1-methylethyl)-								14.08	60.85			14.25
43	Paraldehyde								2.81				
44	13,27-Cycloursan-3-one								27.49				
45	trans-α-Bergamotene							5.54			6.15		
46	Tetracosane							71.91					
47	(3*S*,3a*R*,3b*R*,4*S*,7*R*,7a*R*)-4-Isopropyl-3,7-dimethyloctahydro-1H-cyclopenta[1,3]cyclopropa[1,2]benzen-3-ol							13.15		6.08			
48	Pregnan-3α-ol-20-one									15.83			
49	1-Adamantanemethylamine, α-methyl-												0.72
50	((4a*S*,8*S*,8a*R*)-8-Isopropyl-5-methyl-3,4,4a,7,8,8a-hexahydronaphthalen-2-yl)methanol										3.05		
51	Cyclobarbital										1.05		

**Table 6:** Radical scavenging capacity of DPPH of ethanol, petroleum ether, and ethyl acetate extractives from *Alnus cremastogyne* pods.

Sample	IC_50_ (mg/mL)		
	Ethanol	Petroleum Ether	Ethyl Acetate
A	1.027 ± 0.013 ^e^	3.223 ± 0.010 ^g^	2.405 ± 0.022 ^e^
B	0.513 ± 0.009 ^f^	3.760 ± 0.048 ^e^	2.131 ± 0.023 ^f^
C	0.342 ± 0.001 ^g^	3.388 ± 0.048 ^f^	1.074 ± 0.016 ^h^
D	0.076 ± 0.002 ^i^	1.522 ± 0.031 ^i^	0.390 ± 0.006 ^i^
E	1.379 ± 0.008 ^c^	4.700 ± 0.064 ^c^	2.640 ± 0.034 ^c^
F	1.451 ± 0.078 ^b^	4.904 ± 0.081 ^b^	2.972 ± 0.042 ^b^
G	1.358 ± 0.020 ^cd^	3.387 ± 0.056 ^f^	2.377 ± 0.031 ^e^
H	1.341 ± 0.012 ^cd^	3.387 ± 0.005 ^f^	2.020 ± 0.025 ^g^
I	1.320 ± 0.020 ^d^	3.933 ± 0.060 ^d^	2.516 ± 0.020 ^d^
J	0.184 ± 0.003 ^h^	2.456 ± 0.045 ^h^	0.333 ± 0.006 ^j^
K	1.992 ± 0.026 ^k^	4.975 ± 0.048 ^b^	3.283 ± 0.041 ^a^
L	1.024 ± 0.012 ^e^	5.266 ± 0.087 ^a^	1.980 ± 0.021 ^g^
Vitamin C	0.002 ± 0.000		

Different letters followed the mean values in the same column means significant difference at 0.05 level (*p* < 0.05).

## Data Availability

The data presented in this study are available on request from the corresponding author.

## Sample Availability

Samples of the compounds are not available from the authors.
